# CosinorPy: a python package for cosinor-based rhythmometry

**DOI:** 10.1186/s12859-020-03830-w

**Published:** 2020-10-29

**Authors:** Miha Moškon

**Affiliations:** Faculty of Computer and Information Science, University of Ljubljana, Večna pot 113, 1000 Ljubljana, Slovenia

**Keywords:** Cosinor, Rhythmicity analysis, Circadian analysis, Regression, Python

## Abstract

**Background:**

Even though several computational methods for rhythmicity detection and analysis of biological data have been proposed in recent years, classical trigonometric regression based on cosinor still has several advantages over these methods and is still widely used. Different software packages for cosinor-based rhythmometry exist, but lack certain functionalities and require data in different, non-unified input formats.

**Results:**

We present CosinorPy, a Python implementation of cosinor-based methods for rhythmicity detection and analysis. CosinorPy merges and extends the functionalities of existing cosinor packages. It supports the analysis of rhythmic data using single- or multi-component cosinor models, automatic selection of the best model, population-mean cosinor regression, and differential rhythmicity assessment. Moreover, it implements functions that can be used in a design of experiments, a synthetic data generator, and import and export of data in different formats.

**Conclusion:**

CosinorPy is an easy-to-use Python package for straightforward detection and analysis of rhythmicity requiring minimal statistical knowledge, and produces publication-ready figures. Its code, examples, and documentation are available to download from https://github.com/mmoskon/CosinorPy. CosinorPy can be installed manually or by using pip, the package manager for Python packages. The implementation reported in this paper corresponds to the software release v1.1.

## Background

Many biological processes display oscillations that are under the control of different biological clocks. For example, circadian clocks display daily oscillations, i.e., with a periodicity of approximately 24 h [1], and may regulate nearly half of all genes in a genome of an organism [2, 3]. Disrupted circadian rhythms might have several health implications, such as cardiovascular diseases, diabetes, and immune deficiencies [4]. Analysis of circadian data, especially in the combination with different -omics approaches, thus increases our understanding of disease occurrence and progression. A vast amount of research has been devoted to the analysis of circadian rhythms in recent years [5]. We should as well strive towards the integration of such analyses into the clinical work for disease diagnostics, treatment, and prevention [6].

Detection and analysis of rhythmicity requires designated computational approaches. These approaches are focused on the identification of rhythmic datasets that correspond to specific biological entities (e.g., genes) and evaluation of their rhythmicity parameters. Several non-parametric methods for circadian data analysis have been proposed recently, such as JTK CYCLE and its extensions [7–9] and RAIN [10]. Even though non-parametric methods have several benefits, e.g., robustness to noise in the data, classical harmonic regression should still be used when rhythmicity parameters, such as oscillation amplitudes and acrophases, need to be evaluated, or when the noise in the data is non-Gaussian [10]. Moreover, non-parametric methods often fail or do not perform well when data are (1) collected at irregular intervals, (2) without replicates, (3) unbalanced (i.e., more samples are collected at one time of a day compared to others), (4) full of outliers, and (5) very large. However, cosinor has been successfully applied even in such cases (see, e.g., [11–13]).

Cosinor presents a fundamental method for rhythmicity detection and analysis using cosine curve fitting [14, 15]. It is based on a trigonometric regression model

1 $$\begin{aligned} y(t) = \sum _{i=1}^{N} \left( A_{i,1} \cdot \sin \left( \frac{t}{P/i} \cdot 2 \pi \right) \right. + \left. A_{i,2} \cdot \cos \left( \frac{t}{P/i} \cdot 2 \pi \right) \right) + M + e(t), \end{aligned}$$

where *t* corresponds to the observed time points in the time series, *N* is the number of components in the model, namely number of cosine curves, and $A_{i,1}$, $A_{i,2}$, *C* and *P* are the parameters of the model, with *M* being the MESOR (Midline Statistic Of Rhythm), *P* the period of the observed rhythm, and *e*(*t*) the error term [15]. When the period is known the model can be converted to a linear regression model

2 $$\begin{aligned} y(t) = \sum _{i=1}^{N} \left( A_{i,1} \cdot x_{i,1} + A_{i,2} \cdot x_{i,2}\right) + M+e(t), \end{aligned}$$

where $x_{i,1} = \sin \left( \frac{t}{P/i} \cdot 2 \pi \right)$ and $x_{i,2} = \cos \left( \frac{t}{P/i} \cdot 2 \pi \right)$. When the period is not known, different periods within the feasible period ranges can be tested, or period detection methods such as periodograms, can be used for period estimation [14].

Cosinor has been widely applied to the analysis of rhythmicity detection and evaluation of rhythmicity parameters in time series data. Different software packages for cosinor-based rhythmometry, such as cosinor [16], cosinor2 [17], and DiscoRhythm [18], have been introduced in recent years. However, these packages lack certain functionalities, such as multi-component cosinor regression and analysis, and do not support automatic identification of the best regression model. Moreover, the user must format the input data for each of these tools in a different manner. Herein, we describe CosinorPy, a Python package that merges and extends the functionalities of existing software packages. CosinorPy can as well be used to generate synthetic data, and supports different input and output formats compatible with other software packages for rhythmicity detection and analysis. It provides all functionalities required for the analysis of rhythmic data from data import and preprocessing to removal of outliers, identification of oscillation periods, assessment of the most suitable models and their statistics, analysis of differential rhythmicity, and data plotting, reporting, and export. Moreover, it implements functions to estimate the required number of samples to obtain the results with a predefined statistical significance and can thus as well be used to guide experimental work [19]. CosinorPy is currently the only cosinor package that is implemented in Python, which has become increasingly important in data science, as well as in the field of bioinformatics in recent years. A comparison of features of currently available cosinor-based software packages is provided in Table 1.

**Table 1:** Currently available software packages for rhythmicity detection and analysis based on cosinor method and its extensions

Package	Language	Differential rhythmicity	Linear regression	Non-linear regression	Multiple components	Count data	Population-mean	Design of experiments	References
CosinorPy	Python	Yes	Yes	Yes	Yes	Yes	Yes	Yes	
Cosinor	R	Yes	Yes	No	No	No	No	No	[16]
Cosinor2	R	Yes	Yes	No	No	No	Yes	No	[17]
DiscoRhythm	R	No	Yes	No	No	No	No	No	[18]
LimoRhyde	R	Yes	Yes	No	No	No	No	No	[22]
CircaCompare	R	Yes	No	Yes	No	No	No	No	[23]

*Package*: package name; *language:* the programming language that is used in a combination with a specific package; *differential rhythmicity*: support for the assessment of a differential rhythmicity among two groups of measurement; *linear regression* and *non-linear regression*: what kind of regression the package supports; *multiple components*: support for multi-component cosinor analysis; *count data*: support for count data analysis; *population-mean*: support for population-mean cosinor analysis; *design of experiments*: support for design of experiments to approximate the minimal number of required samples; *reference*: reference to the package

## Implementation

CosinorPy is implemented in Python and relies on the state-of-the-art Python packages for data management, scientific computing, visualisation, and statistical modelling, namely pandas, NumPy, SciPy, Matplotlib, and statsmodels. CosinorPy is comprised of three Python modules, namely file_parser, cosinor, and cosinor1. The file_parser module implements reading and writing of *xlsx* and *csv* files and generating synthetic data. The cosinor module implements the functionalities based on a single- or multi-component cosinor model that include model fitting, identification of the most suitable model, and analysis of differential rhythmicity. The cosinor1 module implements similar functionalities, which are adapted to a single-component cosinor model, and thus provide more exhaustive results and additional statistics such as the significance of acrophase shifts within the differential rhythmicity analysis. The implementation of specific functionalities within the package are described below. Thorough documentation of the package is available at https://github.com/mmoskon/CosinorPy/blob/master/docs/docs.md.

### Single-component cosinor

When the observed data can be accurately described with a single harmonic component, a single-component cosinor model can be used:

3 $$\begin{aligned} y(t) = A_1 \cdot x_1 + A_2 \cdot x_2 + M + e(t). \end{aligned}$$

Using this model, amplitude (*A*) and acrophase ($\phi$) can be estimated directly from the assessed parameter values as:

4 $$\begin{aligned} A = \sqrt{A_1^2 + A_2^2} \end{aligned}$$

and

5 $$\begin{aligned} \phi = {\left\{ \begin{array}{ll} - \arctan \left( \left| \frac{A_1}{A_2}\right| \right) &{} A_1> 0, A_2> 0,\\ - \pi + \arctan \left( \left| \frac{A_1}{A_2}\right| \right) &{} A_1> 0, A_2< 0,\\ - 2\pi + \arctan \left( \left| \frac{A_1}{A_2}\right| \right) &{} A_1< 0, A_2 > 0,\\ - \pi - \arctan \left( \left| \frac{A_1}{A_2}\right| \right) &{} A_1<0, A_2 < 0. \end{array}\right. } \end{aligned}$$

The statistical significance of the model is assessed with an F-test on the basis of the model sum of squares and sum of square residuals [15]. The significance of rhythmicity is evaluated with the zero amplitude test, which is as well based on the F-statistic [15, 19]. Moreover, period and acrophase significance and confidence intervals are assessed directly from the model and its underlying data [19]. The adequacy of the model can be assessed using different regression diagnostic tests. When replicates are available or when the measurements are performed for several periods, the goodness of fit of the model is evaluated with an F-test comparing the pure error and the lack of fit sum of squares [15].

When collecting circadian data, experimentalists should follow specific guidelines to obtain statistically significant results [20]. However, if certain requirements regarding the precision of the assessment of rhythmicity parameters, e.g. a maximal acceptable length of a confidence interval, can be specified in advance, we can approximate the minimal sample size necessary to achieve such precision [19]. CosinorPy implements these functionalities and can thus be used as well during a design of experiments.

### Multi-component cosinor

When a single-component cosinor model is not able to describe our data satisfactorily, e.g., when the goodness of fit test rejects the model, a multi-component cosinor model can be considered [15]. A multi-component cosinor model is able to describe more complex oscillatory dynamics, e.g. peak asymmetry or multiple peaks within one period, which cannot be described with a single harmonic component. Rhythmicity parameters cannot be calculated analytically from this model, but are evaluated from the fitted curve.

Additional components will always increase a model’s accuracy, but on the account of a reduced number of degrees of freedom. This might cause the over-fitting of a model to the observed data. Automatic selection of the best model regarding the number of components is performed using the extra sum-of-squares F-test:

6 $$\begin{aligned} F = \frac{\frac{SSR_1 - SSR_2}{SSR_2}}{\frac{DoF_1 - DoF_2}{DoF_2}}, \end{aligned}$$

where $SSR_1$ and $SSR_2$ present the sum of squared residuals (SSR) for a simpler and a more complex model, respectively, and where $DoF_1$ and $DoF_2$ present the degrees of freedom (DoF) of a simpler and a more complex model, respectively. The more complex model, i.e., the model with a smaller DoF, is selected as more appropriate when the obtained p-value is lower than a predefined threshold. Moreover, the model selection process can be guided with the goodness of fit measures as for a single-component cosinor model.

Additional advantage of our implementation of the multi-component cosinor regression is that it allows the user to fit a cosinor model to count data. Here, a generalised Poisson model with a logarithmic link can be used in a combination with a cosinor model to handle over- as well as under-dispersed data [21]. Moreover, CosinorPy allows the user to as well select Poisson or negative-binomial models for the analysis of rhythmicity of count data.

### Population-mean models

When dealing with at least three individuals, and when each individual produces a series of dependant measurements which can be used to establish the individual’s cosinor model, a population-mean cosinor should be used [15]. In this case a cosinor model is fitted to each individual. The response of the whole population is described and analysed as a mean of all individual cosinor models (population-mean cosinor). When using a single-component population-mean cosinor, confidence intervals of rhythmicity parameters are assessed as described in [19]. Moreover, a p-value for the null hypothesis of the amplitude of oscillations being zero is evaluated with an F-test for a single-component population-mean cosinor as described in [15]. The statistical significance and the goodness of fit of a single- or multi-component population-mean cosinor model are assessed in a similar way as for the basic cosinor models [15].

### Analysis of differential rhythmicity

Cosinor models can be used to assess the difference in the rhythmic response of two groups of measurements. Each group either corresponds to a different variable (e.g., two different genes) or to the same variable in different conditions (e.g., the same gene before and after a perturbation). We are usually interested in amplitude changes and acrophase shifts between the groups. Several different methods, which we use in our implementation, have been proposed to assess these differences.

If the data describing both groups can be modelled with single-component cosinor models, a single-component cosinor can as well be used to assess the differential rhythmicity of these two groups. This model is implemented as

7 $$\begin{aligned} y(t) = \left( A_{1,a} + g \cdot A_{1,b}\right) \cdot x_1 + \left( A_{2,a} + g \cdot A_{2,b}\right) \cdot x_2 + M_a + g \cdot M_b + e(t), \end{aligned}$$

where *g* equals 0 if the data belong to the group *a*, and 1 if the data belong to the group *b*. Based on the assessed parameter values, we can estimate the acrophases and amplitudes of each of the groups, as well as the differences between these values and their significance [19]. Moreover, a population-mean single-component cosinor model is adapted to analyse the differential rhythmicity in a similar way [17, 19].

LimoRhyde [22] presents a similar approach that uses a cosinor model and can be adapted to use an arbitrary number of components in the following form

8 $$\begin{aligned}y(t) = \sum _{i=1}^{N} \left( \left( A_{i,1,a} + g \cdot A_{i,1,b}\cdot \right) \cdot x_{i,1} + \left( A_{i,2,a} + g \cdot A_{i,2,b}\right) \cdot x_{i,2}\right) + M_a + g \cdot M_b + e(t). \end{aligned}$$

The significance of each parameter in this model is assessed using a T-test, where the null hypothesis is that a parameter equals zero. When this hypothesis is rejected for any of the rhythmicity parameters of the group *b*, namely $A_{i,1,b}$ or $A_{i,2,b}$, these two groups should reflect differential rhythmicity. While a single-component cosinor can be used to assess the significance of acrophase shift and amplitude change, LimoRhyde is only able to assess whether the difference in rhythmicity between the groups is significant or not.

Non-linear regression might as well be used to evaluate the differential rhythmicity parameters and their confidence intervals as described in CircaCompare [23]. This is implemented as the following model

9 $$\begin{aligned} y(t) = (A_a + A_b \cdot g) \cdot \cos \left( \frac{t}{P} \cdot 2\pi - (\phi _a + \phi _b \cdot g)\right) + M_a + M_b \cdot g + e(t), \end{aligned}$$

where $A_a$, $\phi _a$ and $M_a$ present the amplitude, acrophase, and MESOR of the group *a*, respectively, and $A_a + A_b$, $\phi _a + \phi _b$ and $M_a + M_b$ present the amplitude, acrophase, and MESOR of the group *b*, respectively. CosinorPy as well implements differential rhythmicity assessments based on non-linear regression. However, this approach does not provide any additional information to the approach described in Equation 7.

## Results

We demonstrate the application of selected CosinorPy functionalities on two typical case studies using four groups of synthetically generated time series data with the attributes presented in Table 2. The whole analysis is available as interactive Python notebooks (IPYNB) at https://github.com/mmoskon/CosinorPy.

**Table 2:** Parameters for synthetically generated data

Name	Components	Sampling time	Sampling period	Acrophase	Noise amplitude
Test1	1	48 h	2h	0	0.5
Test2	1	48 h	2h	$\pi$	0.5
Test3	3	48 h	1h	0	0.5
Test4	3	48 h	1h	$\pi$	0.5

Rhythmicity periods of the generated data were set to 24 h in all scenarios. Each scenario had three replicates.

### Case study 1: independent measurements

**Table 3:** Summary of the differential rhythmicity assessment for the first case study

Test	*q* amplitude change)	*q* acrophase shift)
Test1 vs. test2	0.372013	0
Test3 vs. test4	0.372013	0

The differential rhythmicity was assessed using a single-component cosinor. We are usually interested in the significance of amplitude changes, as well as acrophase shifts. When multiple tests are performed, CosinorPy adjusts the significance values using the false discovery rate (FDR) method (reported as *q*-values). The results here indicate that while the amplitude changes are not significant, acrophase shifts are ($q < 0.05$)

**Fig. 1:**
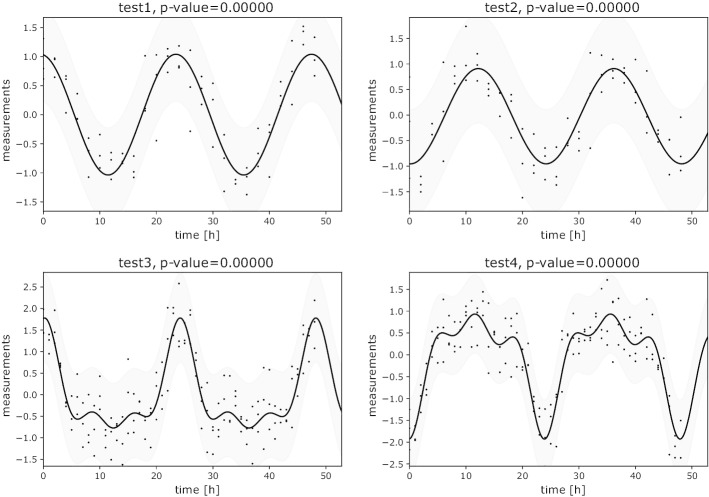
Multi-component cosinor models obtained with the automatic identification of the best fitting models. 1-component models are selected for *test1* and *test2* and 3-component models for *test3* and *test4*. *P* values correspond to the statistical significance of each cosinor model. CosinorPy is able to produce publication-ready figures illustrating the fit of a cosinor model to time series data

In our first case study, we presume that the measurements in each group are independent. This scenario complies with a transcriptomics data analysis or an analysis of qPCR data. CosinorPy successfully identifies the most suitable model, namely a 1-component model in the first two scenarios and a 3-component model in the last two scenarios. If the rhythmicity period is not known, the user could as well use the automatic identification of the best fitting period together with the best fitting model, or could rely on the period values assessed using periodograms (see https://github.com/mmoskon/CosinorPy/blob/master/demo_independent.ipynb). Results obtained with the multi-component cosinor regression are presented in Fig. 1.

**Fig. 2:**
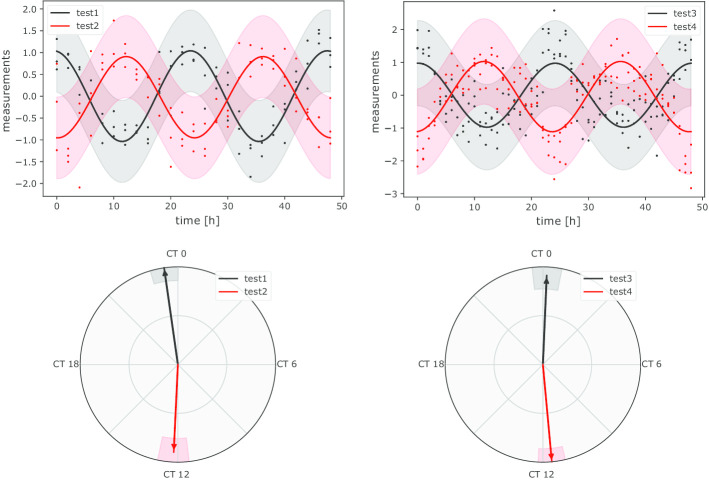
Differential rhythmicity analysis performed with the 1-component cosinor analysis. While the amplitudes are not changed significantly, acrophases differ significantly in both tests (see Table 3). CosinorPy is able to produce publication-ready figures illustrating a comparative analysis of time series data (upper part of the figure), as well as an analysis of acrophase shifts (the lower part of the figure)

Even though a 3-component model is more appropriate for the last two scenarios, a good fit is obtained as well with a 1-component model with slightly higher SSR values than a 3-component model (see Additional file 1: Table 1 and Additional file 2: Table 2). We thus opted to perform a differential rhythmicity analysis using a 1-component model to obtain more informative results, namely the significance of amplitude change and acrophase shift. CosinorPy is able to produce different plots visualising the difference between fits (see the upper part of Fig. 2), as well as acrophase shifts in a polar coordinate system (see the lower part of Fig. 2). Moreover, results of the analysis are reported in a tabular form, i.e., as a pandas DataFrame, which can be easily stored to Excel or CSV format. These results are available in Additional file 3: Table 3 and summarised in Table 3.

**Table 4:** Summary of the differential rhythmicity assessment for the second case study (population-mean cosinor)

Test	*q* (amplitude change)	*q* (acrophase shift)
Test1 vs. test2	0.537841	0.000036
Test3 vs. test4	0.544973	0.000120

The differential rhythmicity was assessed using a single-component population-mean cosinor. When multiple tests are performed, CosinorPy adjusts the significance values using the false discovery rate (FDR) method (reported as *q*-values). The results of the analysis indicate that while the amplitude changes are not significant, acrophase shifts are ($q < 0.05$)

**Fig. 3:**
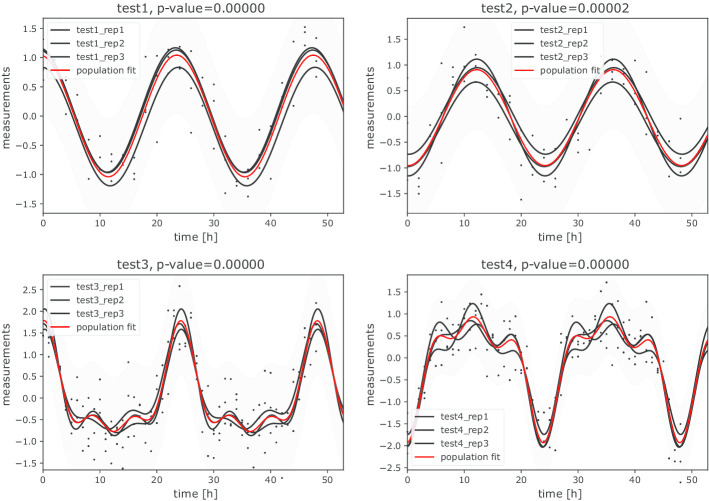
Multi-component population-mean cosinor models obtained with the automatic identification of the best fitting models. 1-component models are selected for *test1* and *test2* and 3-component models for *test3* and *test4*. *P* values correspond to the statistical significance of each cosinor model. Black lines represent the cosinor models of each individual and red lines population-mean cosinor models

The same data were used in a combination with the cosinor and cosinor2 R packages [16, 17] to validate the obtained results. These two packages support only single-component cosinor analyses. Moreover, the cosinor2 package builds upon the cosinor package, which unfortunately reports incorrect acrophase values [17]. Even though the cosinor2 package provides a function to correct these values, their corresponding p-values are not updated accordingly. The analyses performed with the CosinorPy package produce the same results as cosinor and cosinor2 packages with the above mentioned exception (see Additional file 7: Table 7 and Additional file 8: Table 8).

### Case study 2: population-based measurements

In our second case study, we presume that measurements in each group belong to the same individual, which means that population-mean models should be used. This complies with, e.g., bioluminescence data, where the same cell is observed throughout the whole experiment. We can also refer to such measurements as dependent measurements.

We again use CosinorPy to identify the most suitable model, and assess the rhythmicity parameters and significance of periodicity in the data (see https://github.com/mmoskon/CosinorPy/blob/master/demo_dependent.ipynb). As in the first case study, CosinorPy is able to identify the most suitable model for each dataset (see Fig. 3). Complete results of the fitting process are available as Additional file 4: Table 4 and Additional file 5: Table 5. We again opted to use a single-component cosinor to perform the comparison analysis. Results of this analysis are available in Additional file 6: Table 6 and summarised in Table 4.

We validated the obtained results using the cosinor2 R package [17]. The population-based tests implemented within this package do not rely on the cosinor R package. The reported acrophases and their corresponding p-values thus fully comply with the results obtained with the CosinorPy package (see Additional file 9: Table 9 and Additional file 10: Table 10).

### Case study 3: additional benefits of the multi-component cosinor

To additionally investigate the benefits of multi-component cosinor models we applied CosinorPy to a larger dataset downloaded from the JTK Cycle repository [7]. We analysed these data with both, single-component cosinor, as well as multi-component cosinor models with up to three components (see https://github.com/mmoskon/CosinorPy/blob/master/multi_vs_single.ipynb). Among 250 measurements 95 measurement were identified to be circadian using multi-component cosinor models. Among these, 11 measurement were not identified to be circadian using a single-component cosinor model. In all these 11 cases data reflected multiple peaks within a 24-hour period, which cannot be fitted with a single-component model. Multiple peaks were successfully incorporated into multi-component models. However, in some of these cases the statistical significance was marginal, and additional data should be collected to confirm the circadian nature of observed measurements. In the future, large-scale analyses that were performed with single-component cosinor models in the past should be revised using multi-component cosinor models. This could enable us to detect additional rhythmic genes and would thus provide novel insights into circadian dynamics of selected genes.

## Conclusion

CosinorPy provides all the functionalities required for a rhythmicity analysis of experimental data. Its features merge and extend the functionalities of existing cosinor-based software packages. These range from data import and pre-processing to identification of the most suitable models, evaluation of rhythmicity parameters and their significance, and assessment of differential rhythmicity between groups of measurements. Moreover, CosinorPy produces publication-ready figures, visualising the results of the fitting process as well as assessed parameter values, e.g., acrophase values in a polar coordinate system. With the vast scope of functionalities, as well as ease of use, the package presents an attractive alternative to other software packages for rhythmicity detection and analysis.

## Availability and requirements

- Project name: CosinorPy
- Project home page: https://github.com/mmoskon/CosinorPy
- Operating system(s): Platform independent
- Programming language: Python
- Other requirements: pandas, Matplotlib, NumPy, SciPy, statsmodels and openpyxl Python libraries
- License: MIT license
- Any restrictions to use by non-academics: none

## Supplementary information

**Additional file 1: Supplementary Table 1**. Results of the fitting process for the first case study using 1-, 2- and 3-component cosinor models with the cosinor module. The results are presented in a CSV format as reported by CosinorPy.

**Additional file 2: Supplementary Table 2**. Results of the fitting process for the first case study using 1-component cosinor models with the cosinor1 module. The results are presented in a CSV format as reported by CosinorPy.

**Additional file 3: Supplementary Table 3**. Results of the comparison analysis for the first case study using 1-component cosinor models with the cosinor1 module. The results are presented in a CSV format as reported by CosinorPy.

**Additional file 4: Supplementary Table 4**. Results of the fitting process for the second case study using 1-, 2- and 3-component cosinor models with the cosinor module. The results are presented in a CSV format as reported by CosinorPy.

**Additional file 5: Supplementary Table 5**. Results of the fitting process for the second case study using 1-component cosinor models with the cosinor1 module. The results are presented in a CSV format as reported by CosinorPy.

**Additional file 6: Supplementary Table 6**. Results of the comparison analysis for the second case study using 1-component cosinor models with the cosinor1 module. The results are presented in a CSV format as reported by CosinorPy.

**Additional file 7: Supplementary Table 7**. Results of the fitting process for the first case study using cosinor and cosinor2 R packages.

**Additional file 8: Supplementary Table 8** Results of the comparison analysis for the first case study using cosinor and cosinor2 R packages.

**Additional file 9: Supplementary Table 9**. Results of the fitting process for the second case study using cosinor2 R package.

**Additional file 10: Supplementary Table 10**. Results of the comparison analysis for the second case study using cosinor2 R package.

## Data Availability

All data generated or analysed during this study are included in this published article and its supplementary information files available at https://github.com/mmoskon/CosinorPy.

## Abbreviations

CSV: Comma separated values
DoF: Degrees of freedom
FDR: False discovery rate
IPYNB: Interactive python notebook
JTK: Jonckheere–Terpstra–Kendall
MESOR: Midline statistic of rhythm
qPCR: Quantitative polymerase chain reaction
RAIN: Rhythmicity analysis incorporating nonparametric methods
SSR: Sum of squared residuals
