# Supplementary material for: CosinorPy: a python package for cosinor-based rhythmometry
Source: BMC Bioinformatics. 2020 Oct 29;21:485. doi: 10.1186/s12859-020-03830-w (PMC7597035; doi:10.1186/s12859-020-03830-w)
Supplement: Supplementary file 1 — Additional file 1: Supplementary Table 1. Results of the fitting process for the first case study using 1-, 2- and 3-component cosinor models with the cosinor module. The results are presented in a CSV format as reported by CosinorPy. [file 12859_2020_3830_MOESM1_ESM.pdf]

test	period	n_compon	p	q	p_reject	q_reject	RSS	R2	R2_adj
test1	24	1	1.11E-16	1.48E-16	0.169409	0.254113	11.51215	0.784755	0.778776
test1	24	2	1.11E-16	1.48E-16	0.102157	0.204313	11.38718	0.787092	0.774926
test1	24	3	1.11E-16	1.48E-16	0.154183	0.254113	10.74565	0.799087	0.781359
test2	24	1	2.22E-16	2.66E-16	0.247727	0.330303	19.73816	0.63115	0.620904
test2	24	2	3.44E-15	3.75E-15	0.305034	0.366041	18.87176	0.647341	0.627189
test2	24	3	2.33E-14	2.33E-14	0.397668	0.433819	18.0005	0.663622	0.633942
test3	24	1	1.11E-16	1.48E-16	2.73E-13	1.64E-12	66.22364	0.51881	0.512127
test3	24	2	1.11E-16	1.48E-16	3.04E-05	9.11E-05	44.88016	0.673895	0.664709
test3	24	3	1.11E-16	1.48E-16	0.054029	0.129669	36.58674	0.734156	0.722763
test4	24	1	1.11E-16	1.48E-16	3.99E-14	4.78E-13	57.21331	0.60059	0.595042
test4	24	2	1.11E-16	1.48E-16	4.64E-06	1.86E-05	38.95773	0.728033	0.720372
test4	24	3	1.11E-16	1.48E-16	0.69187	0.69187	27.47315	0.808208	0.799988

log-likeliho	period(est)	amplitude	acrophase	mesor	ME	resid_SE
-36.1422	24	1.039764	-6.13224	0.002031	0.797114	0.399864
-35.7329	24	1.047858	-6.02741	-0.02021	0.804413	0.403329
-33.5584	24	1.074762	-5.76535	0.021674	0.793244	0.397523
-56.3604	24	0.93211	-3.19715	-0.02253	1.043747	0.523585
-54.6771	24	0.974877	-2.9875	0.04405	1.035565	0.519227
-52.9046	24	0.944199	-2.67303	0.008264	1.026675	0.514503
-149.975	24	0.976145	-0.05241	-0.00046	1.340413	0.678149
-121.381	24	1.121927	-0.02621	0.356556	1.111343	0.56219
-106.364	24	1.276843	-0.05241	0.503533	1.010686	0.511208
-139.226	24	1.071631	-3.03991	-0.04253	1.245893	0.630329
-110.979	24	1.209467	-2.1227	-0.36488	1.035423	0.523784
-85.3081	24	1.431859	-3.03991	-0.49742	0.875808	0.442986
