# Supplementary material for: CosinorPy: a python package for cosinor-based rhythmometry
Source: BMC Bioinformatics. 2020 Oct 29;21:485. doi: 10.1186/s12859-020-03830-w (PMC7597035; doi:10.1186/s12859-020-03830-w)
Supplement: Supplementary file 2 — Additional file 2: Supplementary Table 2. Results of the fitting process for the first case study using 1-component cosinor models with the cosinor1 module. The results are presented in a CSV format as reported by CosinorPy. [file 12859_2020_3830_MOESM2_ESM.pdf]

| test | p | q | amplitude | p(amplitud | q(amplitud | acrophase | p(acrophas | q(acrophas | acrophase\| |
|---|---|---|---|---|---|---|---|---|---|
| test1 | 9.67E-25 | 1.93E-24 | 1.039766 | 4.96E-59 | 1.98E-58 | 0.141459 | 0 | 0 | -0.54033 |
| test2 | 2.55E-16 | 2.55E-16 | 0.932111 | 1.26E-28 | 1.26E-28 | 3.094333 | 2.48E-254 | 3.30E-254 | -11.8195 |
| test3 | 1.34E-23 | 1.79E-23 | 0.976146 | 1.23E-35 | 1.64E-35 | 6.240175 | 0.599345 | 0.599345 | -23.8357 |
| test4 | 2.01E-29 | 8.02E-29 | 1.071633 | 5.17E-49 | 1.03E-48 | 3.233446 | 0 | 0 | -12.3509 |

| period |
| --- |
| 24 |
| 24 |
| 24 |
| 24 |
