# Supplementary material for: CosinorPy: a python package for cosinor-based rhythmometry
Source: BMC Bioinformatics. 2020 Oct 29;21:485. doi: 10.1186/s12859-020-03830-w (PMC7597035; doi:10.1186/s12859-020-03830-w)
Supplement: Supplementary file 3 — Additional file 3: Supplementary Table 3. Results of the comparison analysis for the first case study using 1-component cosinor models with the cosinor1 module. The results are presented in a CSV format as reported by CosinorPy. [file 12859_2020_3830_MOESM3_ESM.pdf]

test	p	q	amplitude1	p(amplitud	q(amplitud	amplitude2	p(amplitud	q(amplitud	d_amplitud
test1 vs. te	7.11E-37	7.11E-37	1.039766	5.81E-44	1.16E-43	0.932111	1.02E-35	1.02E-35	-0.10766
test3 vs. te	1.64E-49	3.29E-49	0.976146	4.11E-38	4.11E-38	1.071633	1.45E-45	2.90E-45	0.095487

p(d_amplit	q(d_amplit	acrophase1	p(acrophas	q(acrophas	acrophase2	p(acrophas	q(acrophas	d_acropha	p(d_acroph
0.308457	0.372013	-6.14173	0	0	-3.18885	0	0	2.952874	1.16E-153
0.372013	0.372013	-0.04301	0.586319	0.586319	-3.04974	0	0	-3.00673	4.81E-174

q(d_acroph	period
1.16E-153	24
9.62E-174	24
