# Supplementary material for: CosinorPy: a python package for cosinor-based rhythmometry
Source: BMC Bioinformatics. 2020 Oct 29;21:485. doi: 10.1186/s12859-020-03830-w (PMC7597035; doi:10.1186/s12859-020-03830-w)
Supplement: Supplementary file 4 — Additional file 4: Supplementary Table 4. Results of the fitting process for the second case study using 1-, 2- and 3-component cosinor models with the cosinor module. The results are presented in a CSV format as reported by CosinorPy. [file 12859_2020_3830_MOESM4_ESM.pdf]

| test | period | n_compon | p | q | p_reject | q_reject | RSS | period(est) | amplitude |
|---|---|---|---|---|---|---|---|---|---|
| test4 | 24 | 1 | 1.11E-16 | 1.48E-16 | 3.99E-14 | 4.78E-13 | 57.21331 | 24 | 1.071631 |
| test4 | 24 | 2 | 1.11E-16 | 1.48E-16 | 4.64E-06 | 1.86E-05 | 38.95773 | 24 | 1.209467 |
| test4 | 24 | 3 | 1.11E-16 | 1.48E-16 | 0.69187 | 0.69187 | 27.47315 | 24 | 1.431859 |
| test2 | 24 | 1 | 2.22E-16 | 2.66E-16 | 0.247727 | 0.330303 | 19.73816 | 24 | 0.93211 |
| test2 | 24 | 2 | 3.44E-15 | 3.75E-15 | 0.305034 | 0.366041 | 18.87176 | 24 | 0.974877 |
| test2 | 24 | 3 | 2.33E-14 | 2.33E-14 | 0.397668 | 0.433819 | 18.0005 | 24 | 0.944199 |
| test1 | 24 | 1 | 1.11E-16 | 1.48E-16 | 0.169409 | 0.254113 | 11.51215 | 24 | 1.039764 |
| test1 | 24 | 2 | 1.11E-16 | 1.48E-16 | 0.102157 | 0.204313 | 11.38718 | 24 | 1.047858 |
| test1 | 24 | 3 | 1.11E-16 | 1.48E-16 | 0.154183 | 0.254113 | 10.74565 | 24 | 1.074762 |
| test3 | 24 | 1 | 1.11E-16 | 1.48E-16 | 2.73E-13 | 1.64E-12 | 66.22364 | 24 | 0.976145 |
| test3 | 24 | 2 | 1.11E-16 | 1.48E-16 | 3.04E-05 | 9.11E-05 | 44.88016 | 24 | 1.121927 |
| test3 | 24 | 3 | 1.11E-16 | 1.48E-16 | 0.054029 | 0.129669 | 36.58674 | 24 | 1.276843 |

| acrophase | mesor | ME | resid_SE |
|---|---|---|---|
| -3.03991 | -0.04253 | 1.245893 | 0.630329 |
| -2.1227 | -0.36488 | 1.035423 | 0.523784 |
| -3.03991 | -0.49742 | 0.875808 | 0.442986 |
| -3.19715 | -0.02253 | 1.043747 | 0.523585 |
| -2.9875 | 0.04405 | 1.035565 | 0.519227 |
| -2.67303 | 0.008264 | 1.026675 | 0.514503 |
| -6.13224 | 0.002031 | 0.797114 | 0.399864 |
| -6.02741 | -0.02021 | 0.804413 | 0.403329 |
| -5.76535 | 0.021674 | 0.793244 | 0.397523 |
| -0.05241 | -0.00046 | 1.340413 | 0.678149 |
| -0.02621 | 0.356556 | 1.111343 | 0.56219 |
| -0.05241 | 0.503533 | 1.010686 | 0.511208 |
