# Supplementary material for: CosinorPy: a python package for cosinor-based rhythmometry
Source: BMC Bioinformatics. 2020 Oct 29;21:485. doi: 10.1186/s12859-020-03830-w (PMC7597035; doi:10.1186/s12859-020-03830-w)
Supplement: Supplementary file 5 — Additional file 5: Supplementary Table 5. Results of the fitting process for the second case study using 1-component cosinor models with the cosinor1 module. The results are presented in a CSV format as reported by CosinorPy. [file 12859_2020_3830_MOESM5_ESM.pdf]

test	p	q	amplitude	LB(amplitu	UB(amplitu	acrophase	LB(acropha	UB(acroph	acrophase[
test1	0.000244	0.000489	1.039766	0.972793	1.10674	-6.14173	-6.33315	-5.97301	23.45967
test2	0.149442	0.149442	0.932111	0.496716	1.367505	-3.18885	-3.25285	-3.11186	12.18052
test3	0.045682	0.060909	0.976146	0.838807	1.113485	-0.04301	-0.25405	0.159531	0.164287
test4	0.000113	0.000453	1.071633	0.747364	1.395902	-3.04974	-3.27513	-2.64511	11.64915

h]
