# Supplementary material for: CosinorPy: a python package for cosinor-based rhythmometry
Source: BMC Bioinformatics. 2020 Oct 29;21:485. doi: 10.1186/s12859-020-03830-w (PMC7597035; doi:10.1186/s12859-020-03830-w)
Supplement: Supplementary file 6 — Additional file 6: Supplementary Table 6. Results of the comparison analysis for the second case study using 1-component cosinor models with the cosinor1 module. The results are presented in a CSV format as reported by CosinorPy. [file 12859_2020_3830_MOESM6_ESM.pdf]

test	amplitude:	amplitude:	p(d_amplit	q(d_amplit	acrophase:	acrophase:	p(d_acropl	q(d_acrophase)
test1 vs te:	1.039766	0.932111	0.26892	0.537841	-6.14173	-3.18885	1.82E-05	3.63E-05
test3 vs te:	0.976146	1.071633	0.544973	0.544973	-0.04301	-3.04974	0.00012	0.00012
