# Supplementary material for: CosinorPy: a python package for cosinor-based rhythmometry
Source: BMC Bioinformatics. 2020 Oct 29;21:485. doi: 10.1186/s12859-020-03830-w (PMC7597035; doi:10.1186/s12859-020-03830-w)
Supplement: Supplementary file 7 — Additional file 7: Supplementary Table 7. Results of the fitting process for the first case study using cosinor and cosinor2 R packages. [file 12859_2020_3830_MOESM7_ESM.pdf]

| test | p | amplitude | p_amplitud | acrophase | p_acropha | acrophase_corrected |
|---|---|---|---|---|---|---|
| test1 | 9.67E-25 | 1.039766 | 4.96E-59 | -0.14146 | 0.0272 | 0.141459 |
| test2 | 2.55E-16 | 0.932111 | 1.26E-28 | 0.04726 | 0.613667 | 3.094333 |
| test3 | 1.34E-23 | 0.976146 | 1.23E-35 | 0.04301 | 0.599345 | 6.240175 |
| test4 | 2.01E-29 | 1.071633 | 5.17E-49 | -0.09185 | 0.185075 | 3.233446 |
