# Supplementary material for: CosinorPy: a python package for cosinor-based rhythmometry
Source: BMC Bioinformatics. 2020 Oct 29;21:485. doi: 10.1186/s12859-020-03830-w (PMC7597035; doi:10.1186/s12859-020-03830-w)
Supplement: Supplementary file 8 — Additional file 8: Supplementary Table 8 Results of the comparison analysis for the first case study using cosinor and cosinor2 R packages. [file 12859_2020_3830_MOESM8_ESM.pdf]

test	p	amplitude1	p_amplituc	amplitude2	p_amplituc	d_amplituc	p_d_ampli	acrophase1	p_acropha
test1 vs. te	7.11E-37	1.039766	5.81E-44	0.932111	1.02E-35	-0.10766	0.308457	-0.14146	0.057987
test3 vs. te	1.64E-49	0.976146	4.11E-38	1.071633	1.45E-45	0.095487	0.372013	0.04301	0.586319

acrophase2	p_acropha	d_acropha	p_d_acrophase
0.04726	0.570433	0.188719	0.091484
-0.09185	0.201954	-0.13486	0.207119
