# Supplementary material for: CosinorPy: a python package for cosinor-based rhythmometry
Source: BMC Bioinformatics. 2020 Oct 29;21:485. doi: 10.1186/s12859-020-03830-w (PMC7597035; doi:10.1186/s12859-020-03830-w)
Supplement: Supplementary file 9 — Additional file 9: Supplementary Table 9. Results of the fitting process for the second case study using cosinor2 R package. [file 12859_2020_3830_MOESM9_ESM.pdf]

| test | p | amplitude | LB_amplitu | UB_amplit | acrophase | LB_acroph | UB_acrophase |
|---|---|---|---|---|---|---|---|
| test1 | 0.000244 | 1.039766 | 0.972793 | 1.10674 | -6.14173 | -6.33315 | -5.97301 |
| test2 | 0.149442 | 0.932111 | 0.496716 | 1.367505 | -3.18885 | -3.25285 | -3.11186 |
| test3 | 0.045682 | 0.976146 | 0.838807 | 1.113485 | -0.04301 | -0.25405 | 0.159531 |
| test4 | 0.000113 | 1.071633 | 0.747364 | 1.395902 | -3.04974 | -3.27513 | -2.64511 |
