# Supplementary material for: CosinorPy: a python package for cosinor-based rhythmometry
Source: BMC Bioinformatics. 2020 Oct 29;21:485. doi: 10.1186/s12859-020-03830-w (PMC7597035; doi:10.1186/s12859-020-03830-w)
Supplement: Supplementary file 10 — Additional file 10: Supplementary Table 10. Results of the comparison analysis for the second case study using cosinor2 R package. [file 12859_2020_3830_MOESM10_ESM.pdf]

test	amplitude1	amplitude2	p_d_ampli	acrophase1	acrophase2	p_d_acrophase
test1 vs. te	1.039766	0.932111	0.26892	-6.14173	-3.18885	1.82E-05
test3 vs. te	0.976146	1.071633	0.544973	-0.04301	-3.04974	0.00012
